# The Influence of Self-Control and Social Status on Self-Deception

**DOI:** 10.3389/fpsyg.2018.01256

**Published:** 2018-09-06

**Authors:** Mengmeng Ren, Bowei Zhong, Wei Fan, Hongmei Dai, Bo Yang, Wenjie Zhang, Zongxiang Yin, Juan Liu, Jin Li, Youlong Zhan

**Affiliations:** ^1^Cognition and Human Behavior Key Laboratory of Hunan Province, Hunan Normal University, Changsha, China; ^2^Department of Psychology, School of Education Science, Hunan Normal University, Changsha, China; ^3^Department of Pediatrics, The Third Xiangya Hospital of Central South University, Changsha, China

**Keywords:** self-deception, forward-looking paradigm, self-control, social status, deception

## Abstract

The purpose of this study was to explore the effects of self-control and social status on self-deception. The present study adopted a forward-looking paradigm to investigate how self-control and social status influence self-deception. In Experiment 1, participants were asked to complete 10 questions, after they predicted and completed 40 questions (commonsense judgment materials) either with or without answer hints. The results indicated that the participants had higher predicted scores under conditions with answer hints compared with conditions without answer hints and that the predicted scores were much higher than the actual scores under conditions with answer hints. In Experiment 2, individuals with different self-control traits were chosen to perform the operation and induction of the perception of social status and then complete tests such as Experiment 1. The results showed that differences in the predicted scores between conditions with answer hints and those without answer hints were observed to be greater in individuals with low self-control traits than in individuals with higher self-control traits, however, such differences between individuals with higher and low self-control traits were only observed in conditions with low social status perception, not in the conditions with high social status perception. The findings indicated that compared with individuals with high self-control, low self-control individuals tended to produce more self-deception. In addition, high social status in the individuals’ perception could restrain the influence of low self-control on self-deception, while low social status in the individuals’ perception could increase the self-control’s influence on self-deception.

## Introduction

The human brain is incredibly powerful, and it can store, encode, and process massive amounts of information, such as time, space, and even multidimensional concepts. However, a natural flaw in the brain is that it sometimes produces a “short circuit” and can send a wrong sense to individuals and cause wrong behavioral decisions. Individuals, however, still believe in the wrong guidance provided by the brain, and this is what we often call “self-deception” (Davidson, 1985). Mitchell (2000) proposed that self-deception refers to the fact that even though there are things that people insist are contrary to the facts, they still adhere to a positive belief in these things. The researchers summarized the previous studies on self-deception and found that self-deception is related to an individual’s sense of happiness, self-esteem, self-confidence, emotional memory, self-service bias, social thinking and altruistic behavior (Erez et al., 1995; Linton and Wiener, 2001; Mele, 2006; Lopez and Fuxjager, 2012; Lynn et al., 2014). Therefore, if self-deception can be properly used, it can have a positive impact on individuals and society. In contrast, if self-deception is improperly used, the harm is comprehensive at the same time. Evolutionary scientists have speculated from the perspective of biological evolution and suspected that self-deception is acquired to better adapt to society and must be produced in special social interactions and life situations (Pears and David, 1984; Mele, 1997; Bandura, 2011). Trivers (2011) began to study the concept of self-deception in 1976, however, after more than 30 years of exploration, he still posited that the study of self-deception was immature. The empirical research of self-deception has received extensive attention in the fields of social psychology and economics. Such research provides many important clues for the study of self-deception, making it possible to explore self-deception (von Hippel and Trivers, 2011). In the field of cognitive psychology, self-deception can often be explained by the motivational theory of self-serving bias. Self-serving bias means that individual who are actuated by some motivation often direct their thoughts toward desirable events or outcomes and away from unsatisfactory events or results (Mcallister et al., 2005). When individuals cheat themselves, they can obtain immediate benefits through two ways: self-promotion and self-expression. In previous studies, the researchers arranged for some participants to take tests and gave them the opportunity to cheat on in exams. If the participants received good grades, they would overestimate their scores on the next exams and believe that they would do well even without answers, which was self-deception based on the motivation of self-promotion (Chance et al., 2011). Therefore, self-deception play a very important role in our lives. We wanted to explore the following: What are the factors that affect self-deception, and how do we better view self-deception?

The relationship between self-control and self-deception has not been studied directly in previous studies. However, there are many studies researching the influence of self-control on deception. Self-control plays a great role in the possibility of deception. When people’s self-control is in a lower state, the probability of deception is greater; on the contrary, people with high self-control can limit deception within themselves. For example, Baumeister and Boden (1998) proposed a self-depletion model, in which self-control was suggested to allow individuals to abandon inappropriate responses and select more appropriate responses. When the lack of self-control, individuals are prone to inappropriate reactions, including lying. Kouchaki and Smith (2014) reported that people were more likely to lie and cheat in the afternoon, after they had used a certain amount of self-control for studying and working in the morning. Fan et al. (2016) showed that compared with individuals with high self-control ability, individuals with low self-control traits exhibited more deception and a greater deception tendency; the individuals who had abundant self-control resources were more likely to withstand the temptation of deception. The current study only discusses the relationship between self-control and deception. Although we cannot determine the direct relationship between self-control and self-deception, we would like indirectly to obtain research on the relationship between self-control and self-deception through the relationship between self-control and deception and the relationship between self-deception and deception. These findings provide the basis for our exploration of the relationship between self-deception and self-control.

According to the Social Dominance Theory, there is a certain connection between self-deception and social status. People with high social status are more likely to become targets of deception, so we assumed that self-deception was more likely to occur when lying to people with higher social status compared with people of equal social status (Cummins, 1999). Lu and Chang (2014) also found that participants showed a weaker recognition ability when the teacher worked as an assistant in the tests than when a student worked as an assistant. In other words, when individuals are in a low social status, the probability of self-deception may be higher. When individuals perceive a different social status, the possibility of self-deception may become different.

Previous studies also explored the bi-relationship between social status and self-control. Ferrer and Krantz (1987) found that there is a significant positive correlation between children’s self-control and status awareness. Hassin et al. (2010) proposed that an unrealistic social status would weaken the self-control of the individual. There are also studies showing that compared with individuals with low self-control ability, individuals with higher self-control ability are more willing to obey rules and commands from a person of high social status. Therefore, individuals with higher self-control ability more easily to achieve career success as well as gain higher social status (Tangney et al., 2004). Studies have found that successful celebrities have a higher social status and self-discipline than the public. People with a lower social status and weak self-control are more likely to ignore or violate rules (Cummins, 1999). Therefore, individuals have different self-control abilities that are based on their different social statuses. Such research results tell us that high self-controllers have a higher social status, but these studies have not directly told us what role self-deception has in the relationship between self-control and social status.

In conclusion, self-control can affect deception, showing that individuals with high self-control exhibit fewer deceptive behaviors compared to individuals with low self-control levels. Self-deception is also associated with deception, and the purpose of self-deception is to better deceive others in society. In this case, can the level of an individual’s self-control affect the individual’s self-deception behavior? Previous studies have found that individuals are more likely to exhibit self-deception when they are deceiving people of a higher social status rather than deceiving people of the same social status. Other studies have shown that individuals have greater self-deception when in a low social status. In this case, can social status regulate the self-deception of individuals with different self-control abilities?

## Experiment 1

### Purpose and Hypothesis

Experiment 1 aimed to research how self-deception of individuals come into being in the forward-looking paradigm. This study hypothesized that compared to the control group (without answer hints), the answer group (with answer hints) would be affected by the answer to show self-deception, in which participants would predict themselves to have a higher score in the second set of tests.

### Materials and Methods

#### Participants

The experimental procedure was granted ethical approval by the ethics committee of the Institute of Psychology, Hunan Normal University. Sixty nonpsychology-major participants (35 males, 25 females, age 18.85 ± 0.67 years) who came to participate in our experiments were college students in our school. All the participants had no mental deficiencies or color blindness. In addition, they were all right handed without serious physical and mental defects. Prior to this, they have not participated in the Chinese national civil service examinations that were related to the administrative professional ability tests and similar tests. They signed their informed consent to the experiment and were given obtained appropriate remunerations after the experiment. The experiment was divided into two stages of tests: the first stage was the control group (17 males, 13 females), and the second stage was the answer group (18 males, 13 females).

#### Commonsense Judgment Material

Commonsense judgment material that mainly measured the basic social knowledge of participants and the basic ability to use these knowledges of analysis and judgment was selected from the Chinese national civil service examination of the administrative professional ability test system. We selected the middle difficulty of the 50 questions and divided them into two sets of tests: the first test included 10 questions, and the second test included 40 questions (40 questions divided into two parts for experiment 2). After selecting 100 ordinary college students to complete two sets of questions, the scores were analyzed in terms of difficulty and division. The statistical results showed that the average difficulty coefficient of the first set of tests was 0.51, while the average difficulty coefficient of the second set of tests was 0.49. The difference in average difficulty of the two sets of tests was not significant, and the difficulty of the topic was moderate; consequently, it was suitable for college students to complete (see Appendix).

### Procedure

**FIGURE 1:**
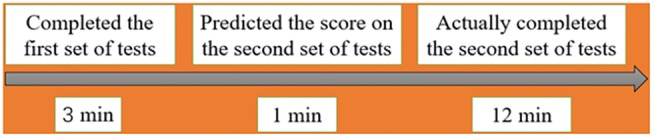
Schematic representation of the forward-looking paradigm in Experiment 1.

Experiment 1 used a single factor design. Answer hints was the independent variable for the experiment, and the dependent variable was the predicted score. At the beginning of the experiment, the control group was asked to complete the first set of tests comprising approximately 10 questions in 3 min (without answer hints) and were told that “the score of the tests can reflect your commonsense judgment ability.” After the first set of tests was completed, the total scores that were calculated by the experimenter were told to the participants. Then, the participants were asked to predict the score obtain though completing the second set of tests (which included 40 questions) in 12 min and to write the predicted score behind the answer sheet. After this, they needed to complete the second set of tests on paper, without any answer hints. The answer group was asked to complete the first set of the same 10 questions in 3 min, while the reference answer could be seen in the bottom right of the paper (with answer hints). Then, the participants were asked to predict the score on the second set of tests and to write it down on the back page of answer sheet. Finally, they were also asked to complete the second set of tests without answer hints (see **Figure 1**).

#### Data Statistics and Analysis

In Experiment 1, we described the score on the first set of tests, the predicted score and the actual score of the second set on tests and the difference between the predicted score and the actual score, from the answer group and the control group. Independent sample *t*-test analysis was used to test the score on the first test, the difference of the predicted score in the two groups, and the difference between the predicted score and the actual score in the two groups.

### Results

#### Statistical Results

**FIGURE 2:**
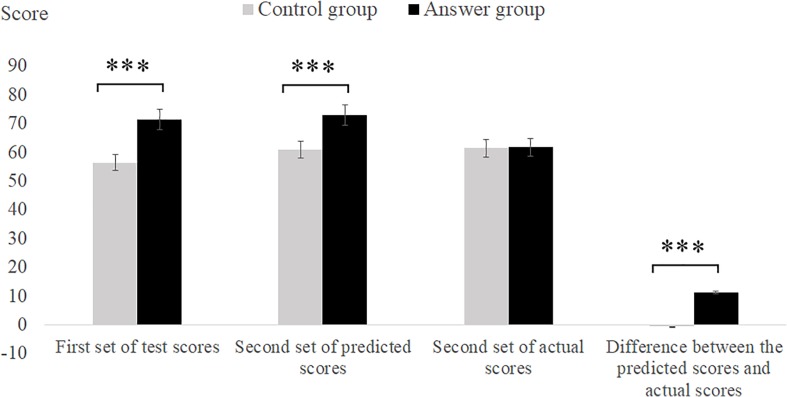
Statistical data of tests scores in Experiment 1. Error bars represent the standard error of the mean. ^∗^*p* < 0.05, ^∗∗^*p* < 0.01, and ^∗∗∗^*p* < 0.001.

The descriptive statistics of the first set of test scores, the second set of test scores, the predicted scores on the second set of tests, and the difference between the predicted score and the actual score on the second set of tests are shown in **Table 1**.The scores in the two groups of the first set of tests were analyzed with an independent sample t-test, and the answer showed that the answer group scores were significantly higher than those of the control group *t*(29) = 7.88, *p* < 0.001]; the predicted scores of the second set of tests in the groups were analyzed with an independent sample *t*-test, and the experiment showed that the answer group’s predicted score was significantly higher than that of the control group [*t*(29) = 6.06, *p* < 0.001], and the actual score on the second set of tests in the answer group was significantly higher than that in the control group [*t*(29) = 4.35, *p* > 0.05]. To further verify whether the answer hints could cause self-deception, we used the independent *t*-test to compute the difference between the two group’s predicted scores subtracted from the actual scores, the experiment showed that the answer group’s predicted scores subtracted from their actual scores were significantly higher than those in the control group [*t*(29) = 5.79, *p* < 0.001] (see **Figure 2**).

**Table 1:** The difference between predicted and actual scores (*M* ± *SD*).

Answer hints	Answer group	Control group
	(*N* = 30)	(*N* = 30)
First set of test scores	71.33 ± 10.42	56.33 ± 8.90
Second set of test scores	61.66 ± 5.45	61.40 ± 4.5
Second set of predicted scores	72.83 ± 8.17	60.83 ± 5.85
Difference between the predicted score and the actual score	11.17 ± 1.39	-0.67 ± 0.45

### Discussion

Experiment 1 showed that participants under conditions with answer hints predicted themselves have higher scores on the second set of tests compared to participants under conditions without answer hints and that the predicted scores were significantly higher than the actual scores. These results were consistent with the previous results. Taylor and Lobel (1989) found that victims of a disaster tend to be unrealistic about themselves, the world or a future fantasy, which creates positive fantasies that can help them cope with threats. Chance et al. (2011) her have found it in their studies that answers have a significant impact on test scores and prompt subjects into self-deception. Their studies indicated that predicted scores of subjects may be influenced by the answers, which makes participants have a higher expected value. Individuals who obtain good scores in conditions with answer hints suggest that the good results reflect their true level. This may be the result of the answer hints. Therefore, these individuals tend to overestimate their ability and underestimate the role of the answer hints, which maybe lead to self-deception.

More importantly, the conclusion of Experiment 1 showed that under conditions with answer hints, the predicted scores of the participants could explain the tendency of self-deception. The difference between the predicted scores and the actual scores could explain the degree of self-deception, and the answer hints can induce self-deception. Therefore, the next experiment used the difference between the predicted score and the actual score as well as the predicted score as the dependent variable to examine the influence of individual factors and social factors on the self-deception behavior.

## Experiment 2

### Purpose and Hypothesis

The purpose of Experiment 2 was to examine the effect of social status on the self-deception of individuals with high and low self-control ability. Compared with an individual’s low self-control ability, an individual’s high self-control ability would yield fewer self-deception behaviors; the low social status of individuals would promote self-deception in individuals who had low self-control, and the high social status of individuals would weaken self-deception in individuals who had low self-control.

### Materials and Methods

#### Participants

The experimental procedure was granted ethical approval by the ethics committee of the Institute of Psychology, Hunan Normal University. In Experiment 2, we distributed self-control scales to 146 college students who were college students in our school to measure their self-control ability. Finally, we successfully recovered 134 copies. The recovery rate was 91.8%. In this measurement, we selected the top 27% of the students as exhibiting high self-control, and then selected the latter 27% of the students as exhibiting low self-control, and each group included 36 participants (43 males, 29 females, *M*_age_ = 18.8 ± 0.75 years). We removed eight participants because they did not describe what they once experienced as “a sense of social status.” Among them, we excluded 4 participants in each of the high and low self-control groups. Finally, in our experiment, there were total 64 participants, which from the high self-control group (20 males, 12 females) and the low self-control group (19 males, 13 females). All participants had no history of mental deficiency or color blindness and were all right handed without serious physical and mental defects. They signed their informed consent to the experiment and were given appropriate remunerations after the experiment.

#### Experimental Material

Tan and Guo (2008) revised the self-control scale for college students with 19 questions in five dimensions. The internal consistency coefficient α coefficient was 0.862, which showed good reliability and validity. The first set of tests was the same as Experiment 1, and another set of tests consisted of two parts; each part included 20 questions for measurement, which was the same as for Experiment 1.

The Commonsense Judgment materials of Experiment 2 were the same as those of Experiment 1.

The social status-inducing materials were as follows: Take Wang et al. (2013) “recall task” methods to induce individuals’ high and low social status. “Recall task” was the method that measured the social status of the participants. The specific operation of this method can be seen in the following content:

High social status: The participants who were assigned to the high social status group were asked, “Please recall your experience. In this experience, you had a higher social status than someone, that is, you could control someone and could control what other people wanted to get. Describe some experiences when you had a high social status, what happened in this matter, how you felt at that time, etc. You will have 4 min to describe–the more detailed, the better.”

“How did you rank your social status in the scenario you describe?” “1” meant “none at all,” and “7” meant “having a very high social status.”

Low social status: The participants who were assigned to the low social status group were asked: “In daily life, there are often experiences that make you feel that you have no social status. For example, applicants meeting with an examiner, students participating in the thesis defense, leaders who are accused, and so on. Please recall your experiences in which you had a lower social status than someone, that is, someone who had the ability to control you or could control what you wanted to get. Please describe what you experienced without any social status, what happened in this matter, how you felt at that time. You will have 4 min to describe–the more detailed, the better.”

“How did you rank your social status in the scenario you describe?” “1” meant “none at all,” and “7” meant “having a very high social status.”

### Procedure

In Experiment 1, we found that there was a phenomenon of self-deception under the answer hints condition, so we used this conclusion directly in Experiment 2 to let participants experiment under conditions with answer hints. This Experiment 2 had a two-factor mixed experimental design for the participants. In Experiment 2, the participants who had either high or low self-control were induced into different social statuses (high and low). Participants who were successfully induced performed the forward-looking paradigms. We divided Experiment 2 in two parts, and before and after each part of the experiment, we had to induce the social status of the participants. In the first part of the test in Experiment 2, we induced a high social status in the high and low self-control groups, and then we tested the participants’ social status. They were asked, “How did you rank your social status in the scenario you described?” “1” meant “none at all,” “7” meant “having a very high social status.” Next, the participants were asked to complete the tests of self-deception, which were the same as those in Experiment 1, and we needed to test the social status of the participants before the tests and after the tests. This protocol was what we call the pretest and the post-test. The second set of tests’ actual score did not inform the participants.

**FIGURE 3:**
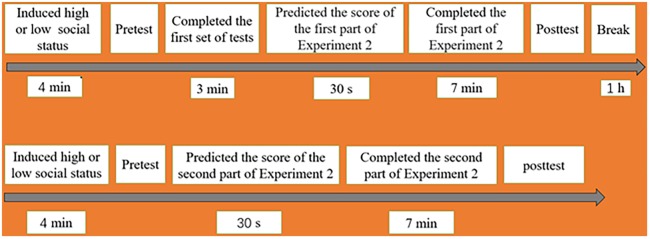
Schematic representation of the forward-looking paradigm under high (up) and low (down) social status in Experiment 2.

After 1 h of rest, we induced a low social status of the participants in the high and low self-control groups to complete the second part of Experiment 2. We pretested the low social status of the participants. Then, all participants were asked to complete the tests of self-deception, which were the same as those in Experiment 1. Then, the experimenters let all participants predict and complete the tests of self-deception, which were the same as those in Experiment 1. Finally, we also post tested the social status of all participants (see **Figure 3**).

### Results

#### Operation Test Check

We analyzed the results of relevant sample t-tests from participants who had an induced high or low social status. We found that the differences in the pretest were extremely significant [*t*(63) = 31.40, *p* < 0.001] with respect to the social status. We also found that compared with the high social status of participants (*M* = 6.02, *SD* = 0.77), the induced scores (*M* = 2.03, *SD* = 0.64) were significantly lower than those in participants with a low social status. The social status was significantly different in post-tests [*t*(63) = 31.84, *p* < 0.001]. Participants with a low social status (*M* = 1.97, *SD* = 0.64) scored significantly lower than those with a high social status (*M* = 6.03, *SD* = 0.76).

#### Predicted Score of the Second Part of Experiment 2

##### Descriptive results

The predicted scores of the first part of the tests that were described by statistical data are shown in **Table 2**.

**Table 2:** Predicted scores of the first part of the tests (*M* ± *SD*).

Social status	Self-control ability	Predicted scores
High	High	62.19 ± 5.67
	Low	63.13 ± 7.04
Low	High	62.97 ± 8.69
	Low	70.63 ± 7.80

##### Results of the variance analysis

**FIGURE 4:**
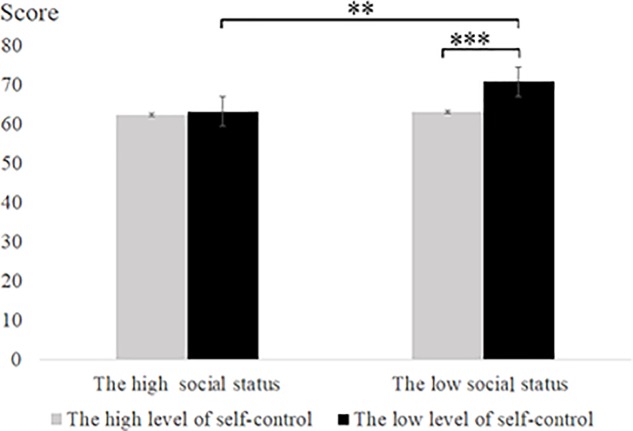
Predicted score on second part of tests in Experiment 2. Error bars represent the standard error of the mean. ^∗^*p* < 0.05, ^∗∗^*p* < 0.01, ^∗∗∗^*p* < 0.001.

The two-factor repeated-measures analyses of variance were performed on the predicted score of the second part of the tests in the answer group. The results showed a significant main effect of social status, and low social status induced higher predicted scores than did high social status [*F*(1,62) = 8.24, *p* < 0.01, $\eta_{p}^{2}$ = 0.02]. There was a significant main effect of self-control ability [*F*(1,62) = 13.89, *p* < 0.001, $\eta_{p}^{2}$ = 0.18], and low self-control subjects predicted higher predicted scores than high self-control participants. There was a significant interaction effect between social status and self-control ability [*F*(1, 62) = 5.42, *p* < 0.05, $\eta_{p}^{2}$ = 0.08]. The simple effect analysis found that for low social status, low self-control participants predicted higher predicted scores than high self-control participants [*F*(1,31) = 13.75, *p* < 0.001]; at the low level of self-control, low social status induced higher predicted scores than high social status [*F*(1,31) = 13.29, *p* < 0.01] (see **Table 3** and **Figure 4**).

**Table 3:** Variance analysis table for the predicted score of the second set tests.

	*F*	*P*	$\eta_{p}^{2}$
Self-control ability	8.24**	0.006	0.02
Social status	13.89***	0.000	0.18
Self-control ability ^∗^Social status	5.42**	0.023	0.08

*^∗^p < 0.05, ^∗∗^p < 0.01, ^∗∗∗^p < 0.001*.

#### Difference Between the Predicted Score and the Actual Score

**Table 4** shows the difference between the actual score and the predicted score in the second part of the tests in Experiment 2 that were described by statistical data.

**Table 4:** Difference between the actual score and the predicted score (*M* ± *SD*).

Social status	Self-control ability	Difference between the actual score and the predicted score
High	High	0.63 ± 0.49
	Low	2.50 ± 1.96
Low	High	2.81 ± 1.61
	Low	10.47 ± 3.36

### Results

**FIGURE 5:**
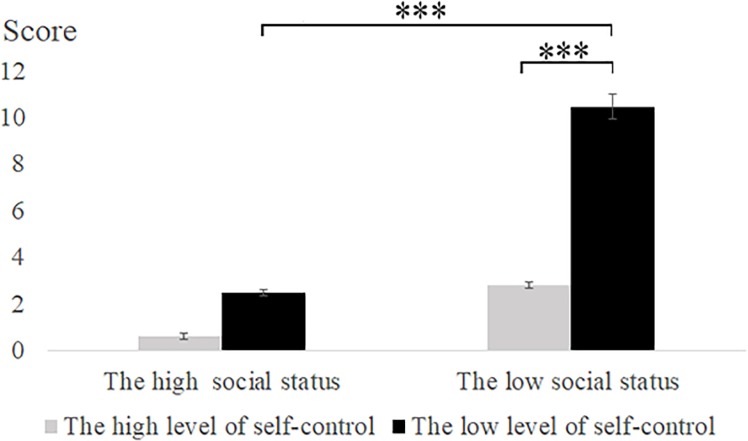
The statistical data of the difference between actual score and predicted score on second part of tests in Experiment 2. Error bars represent the standard error of the mean. ^∗^*p* < 0.05, ^∗∗^*p* < 0.01, ^∗∗∗^*p* < 0.001.

The difference between the predicted score and the actual score in the second part of Experiment 2 was analyzed by two-way repeated measures analysis of variance. The results showed that there was a significant main effect of social status [*F*(1,62) = 22.14, *p* < 0.001, $\eta_{p}^{2}$ = 0.26] and that low social status induced higher scores than high social status. There was a significant main effect of self-control ability [*F*(1,62) = 10.85, *p* < 0.01, $\eta_{p}^{2}$ = 0.15], and low self-control participants predicted higher scores than high self-control participants. There was a significant interaction effect between social status and self-control ability [*F*(1,62) = 7.82, *p* < 0.01, $\eta_{p}^{2}$ = 0.11]. We conducted a simple effect analysis under low social status conditions and found that the difference between the predicted score and the actual score of the low self-control participants was significantly higher than that of the high self-control participants [*F*(1, 31) = 16.66, *p* < 0.001]. We conducted a simple effect analysis under the low self-control ability and found that the difference between the predicted score and the actual score of the low social status participants was significantly higher than that of the high social status participants [*F*(1,31) = 29.17, *p* < 0.001] (see **Table 5** and **Figure 5**).

**Table 5:** Variance analysis table for the difference between the second predicted score and the actual score.

	*F*	*P*	$\eta_{p}^{2}$
Self-control ability	22.14***	0.000	0.26
Social status	10.85**	0.002	0.15
Self-control ability ^∗^Social status	7.82**	0.007	0.11

*^∗^p < 0.05, ^∗∗^p < 0.01, ^∗∗∗^p < 0.001*.

### Discussion

The results of the operation pretest and post-test of social status showed that the scores for the social status perception of participants were significantly higher under the high social status than the low social status. This finding was consistent with the results of previous studies. Previous experimental results have shown a significant difference in the social status perception of participants under a high and low social status (Lammers et al., 2008; Acikalin et al., 2009). The results showed that the manipulation of an individual social status could induce high and low social status effectively.

The study found that compared with individuals with high self-control, the score for the second set of tests of individuals with low self-control were significantly higher, and the difference between the predicted score and the actual score was also significantly higher. This finding was consistent with the results of Experiment 1and showed that compared with participants with a high self-control ability, the participants with a low self-control ability exhibited more self-deception. The results also showed that compared with high social status individuals, low social status individuals had a significantly higher score in predicting the second set of tests, and the difference between the predicted score and the actual score was significantly higher. Previous studies have found that if individuals are confronted with persons who have a higher social status than their social status, it is easier to evade the punishment of the high status by using self-deception, which means that individuals are prone to more self-deception in a low social status (Cummins, 1999). Lu and Chang (2014) found that participants in a task of vocabulary recognition showed a weaker recognition ability when teachers were assistants compared to when students were assistants. These findings suggested that people with a low social status were more likely to cheat themselves. Lopez and Fuxjager (2012) also found that people often used self-deception to improve their social image and status.

As it turns out, under conditions with answer hints, when individuals not only were in a low social status but also had in a low level of self-control ability, their predicted scores were higher than the actual scores. This finding indicated that individuals who were induced into low self-control by the low social status exhibited more blindness and predicted higher scores, ultimately showing had more self-deception behaviors. When people were in a high social status, less self-deception occurred regardless of whether the individual had high self-control or low self-control, which showed that a high social status of individuals promoted the influence of self-control on self-deception. Previous research found not only that individuals with different social statuses differ in their self-control ability but also that such status affects other behaviors of the individuals by directly influencing self-control (Baron, 2003; Gottfredson, 2006). These results suggest that social status might regulate the self-deception of individuals with different self-control abilities.

## General Discussion

### Self-Deception in the Forward-Looking Paradigm

Most previous studies of self-deception used the retrospective paradigm to verify the generation of self-deception through the inconsistencies between the behavioral responses and the subjective reporting (Gur and Sackeim, 1979; Quattrone and Tversky, 1984; Sloman et al., 2010; Pinker, 2011). However, this method cannot always solve the problem of measuring unconsciousness in self-deception. Therefore, there were great disadvantages in the exploration of the production and influencing mechanisms of self-deception. Chance et al. (2011) used the forward-looking paradigm to investigate self-deception and avoid measuring unconscious steps.

Specifically, the present study used the forward-looking paradigm to induce self-deception by adopting Chinese standardized tests, which investigate the rationality and applicability of the model under the influence of oriental culture. The results not only showed that we examined the degree of self-deception of different individuals but also confirmed that when a reliable condition is provided for individuals to deceive themselves, the degree of the individuals’ self-deception will deepen accordingly. Trivers (2000) believed that the individuals in this process exerted self-control and gradually turned conscious information into unconscious information, which meant that self-deception can be regulated. The forward-looking paradigm can ignore the effects of this unconscious information and provide an experimental basis for further research and interpretation of the emergence and development of self-deception.

### Individuals With High Self-Control Know How to Draw on the Advantages and Avoid the Disadvantages of Self-Deception

Self-control is one of the most powerful capabilities of the human mind and benefits the individual. It is the process by which an individual overcomes his own desires and needs to change his or her own behavior and thinking (Tan and Guo, 2008). Experiment 2 showed that under the condition with answer hints, under certain conditions of time and motivation, the degree of self-deception was significantly different both for individuals with high self-control and for individuals with low self-control, which indicated that individuals with low self-control and individuals with high self-control both show a trend toward self-deception. However, compared with the low self-control individuals, high self-control individuals exhibited lower self-deception, which showed that high self-control individuals better knew how to avoid the negative effects of self-deception.

The study of Chance et al. (2015) showed that self-deception produced by individuals in a test may be hidden in the individual’s unconsciousness when self-deception predicted good results. When self-deception produces erroneous results and when wrong results continue to appear, this process may turn into consciousness. Norton et al. (2004) found that individuals with different traits might be consciously deceiving themselves for certain reasons, which suited the self-serving bias theory. Taylor and Lobel (1989) found that the human brain not only can block negative information but also can create positive illusions to help individuals cope with threats, which shows that self-deception is especially suited to a person who is frustrated. In daily life, we occasionally see patients with advanced cancer who exhibit strong self-control to actively believe that their condition is not so serious. Taylor have demonstrated the use of self-deception and self-control to guide and treat patients with depression and mental illness. These studies show that high self-control individuals seem to better understand how to use self-deception to guide behavior profitably.

### High Social Status Can Inhibit the Self-Deception of Individuals With Low Self-control

Experiment 2 showed that under the condition with answer hints, when individuals were in a low social status, self-deception tendencies and self-deception were higher in low self-control individuals than in individuals with high self-control. This finding was basically consistent with those of previous studies, and previous research findings showed that individuals under different social status conditions differ not only in their self-control ability but also in other behaviors by directly influencing self-control (Baron, 2003; Gottfredson, 2006). These results suggest that social status might be able to regulate the self-deception of individuals with different self-control abilities.

Dufner et al. (2013) reported that the use of self-deception to obtain social benefits is often not guaranteed, but also requires certain self-conditions and social conditions. In daily life, high self-control individuals tend to improve their skills and comprehensive qualities by means of hard study to obtain higher social status and social resources. Schmeichel and Vohs (2009) believed that a low self-controller will use self-improvement methods to hide their flaws and improve their positional awareness rather than to gain more practical social resources through actions. In fact, this social status perception is an overhead self-awareness and is also a form of self-deception. Ein-Dor and Perry (2014) believed that this means of obtaining social benefits and high social status through self-deception often drives individual desires and achievements. This process, on the surface, give rise to the purpose of the individual pursuit of social interests–in fact, self-performance and self-improvement to the purpose of self-service. Therefore, the influence of self-control and social status on self-deception was also applied to the motivational theory of self-serving bias.

In sum, individuals with high self-control traits know how to use self-deception to guide behavior profitably, however, individuals with low self-control traits can restrain self-deception behavior at a high social status. In daily life, we might be able not only to use training or punishment to improve self-control among ordinary people but also to promote the social status of others by giving more praise or elevation. We should take the essence and reject the dross so that the value of the self-deception proposition will be further promoted and developed in human society.

## Author Contributions

MR and BZ were responsible for the preparation of experimental procedures and wrote the manuscript. HD and BY analyzed the data. ZY, WZ, and JuL performed the experimental procedures and organized the participants for the experiment. JiL and YZ examined experimental material. WF reviewed the manuscript.

## Conflict of Interest Statement

The authors declare that the research was conducted in the absence of any commercial or financial relationships that could be construed as a potential conflict of interest.

## Appendix

### Commonsense Judgment Material

Dear classmates,

The following is a test that can reflect our ability to judge commonsense judgment. Please answer carefully. After the first tests are completed, you will be asked to predict the score obtain through completing the second set of tests (which includes 40 questions) in 12 min and to write the predict score behind the answer sheet.

### Reference Answer: ACDCC BDCBC

You are asked to predict the score obtain though completing the second set of tests (which includes 40 questions) in 12 min and to write the predicted score behind the answer sheet: ________.
